# Study on the Vibration Isolation Mechanism of Loofah Sponge

**DOI:** 10.3390/biomimetics10010005

**Published:** 2024-12-26

**Authors:** Weijun Tian, Xu Li, Xiaoli Wu, Linghua Kong, Naijing Wang, Shasha Cao

**Affiliations:** 1 Key Laboratory of Bionic Engineering, Ministry of Education, Jilin University, Changchun 130022, China; tianweijun@jlu.edu.cn (W.T.); xuli23@mails.jlu.edu.cn (X.L.); xlwu24@mails.jlu.edu.cn (X.W.); klh1234562024@163.com (L.K.); 2 Siping Heat Exchange Product Quality Inspection Center, Siping 136000, China; 3 Wuhan Institute of Shipbuilding Technology, Wuhan 430050, China

**Keywords:** loofah sponge, energy absorption, vibration isolation

## Abstract

The loofah sponge has a complex, three-dimensional, porous mesh fiber structure characterized by markedly low density and excellent vibration isolation properties. In this study, loofah sponges made from dried *Luffa cylindrica* were divided into two components: the core unit and the shell unit, which were further subdivided into five regions. Static compression performance tests and vibration isolation analysis were conducted on the loofah sponge and its individual parts. Scanning models of the loofah sponge were generated using the RX Solutions nano-CT system in France, and finite element analysis was performed using the ANSYS Workbench. This study focused on the vibration isolation performance of the loofah sponge, examining energy absorption and isolation, as well as the vibrational strength of its isolation performance. The goal was to explore the functions and vibration isolation mechanisms of its different components. The results demonstrated that the loofah sponge structure exhibits rigid–flexible coupling, with the coordinated action of multiple parts producing highly effective energy absorption and isolation of the vibration intensity effect. Specifically, the core unit of the loofah sponge provides the best isolation effect of axial vibration intensity, with an acceleration vibration transfer of −60 dB at 300 Hz. Furthermore, both the core and shell unit structures combine to provide multidirectional low-frequency vibration isolation. This study of the loofah sponge’s vibration isolation mechanism provides a theoretical foundation and new insights for the design of bionic low-frequency vibration isolation devices.

## 1. Introduction

After the *Luffa cylindrica* matures and naturally dries, the remaining three-dimensional porous network fibrous framework structure—formed after removing the outer skin and seeds—is known as the loofah sponge. The fruit of the loofah (*Luffa cylindrica*) typically has a long, spindle-shaped appearance. The structure of the loofah sponge is similar to that of a porous material, and it is commonly referred to as a natural sponge, making it a sustainable biological material [1,2,3,4,5]. This structure arises because, during the growth of the loofah sponge, the cellulose, hemicellulose, and lignin within the fruit intertwine to form a complex three-dimensional porous network of fibers. Additionally, the high cellulose content (up to 60–70%) in the loofah sponge fibers contributes to the stiffness and toughness of its three-dimensional porous structure [6,7,8]. The loofah sponge’s structure is the result of long-term evolutionary adaptation to tropical and subtropical environments, often characterized by complex climatic conditions, such as strong winds and precipitation [9,10]. As the loofah sponge matures and the skin forms, the flesh disappears, and the quality diminishes due to water evaporation. The loofah sponge becomes more vulnerable to external vibration sources, such as wind, rain, and animal activity, which can cause the seeds to fall out. Therefore, the loofah sponge has evolved a multilayered, coupled three-dimensional porous network structure to adapt to mechanical stress and vibration environments to protect the fruit and seeds. This coupled porous three-dimensional network layered structure not only reduces the mass of the loofah sponge skin but also helps absorb external impacts. When subjected to external impacts and vibrations, part of the energy is converted into heat through internal friction and airflow within the pores. Furthermore, vibration waves are reflected and scattered multiple times as they pass through the sponge, ultimately weakening or dissipating. This process absorbs external vibrational energy and isolates the seeds from the intensity of the vibrations.

Loofah sponge, a natural biological material, has attracted significant attention in various research fields in recent years, particularly for its unique advantages in buffering energy absorption and as an environmentally friendly reinforcing agent in composite materials [11,12,13]. The mechanical properties of loofah sponge are primarily attributed to its porous, fibrous structure. Studies have shown that Young’s modulus and the strength of its individual fibers are comparable to those of wood, and the material exhibits strong adsorption properties [14,15]. Over millions of years of evolution, *Luffa cylindrica* has developed a porous three-dimensional architecture with mechanical rigidity similar to keratosan [16]. Its excellent elasticity and strong adsorption properties make this material a promising candidate for use as a catalyst support [17]. Su et al. examined the honeycomb-like porous structure of loofah sponge and developed a new type of highly efficient phase change material with high encapsulation capacity and reliability, capable of stably storing and releasing energy [18]. Al-Mobarak et al. improved the mechanical properties of loofah sponge fibers through chemical treatments, significantly improving their tensile strength and thermal stability. This research provides new insights into the potential applications of high-performance composite materials derived from loofah sponge fibers [19]. Liu et al. developed a multilayer porous carbon material derived from loofah sponge for microwave absorption applications. Their study showed that the sponge-derived carbon composite material achieved excellent impedance matching and efficient microwave absorption properties by combining magnetic nanoparticles with a porous carbon substrate, reaching a maximum absorption value of −43.8 dB at an X-band frequency of 8.3 GHz [20]. Research on the applications of loofah sponge structures has primarily focused on light industry. Owing to its natural multidirectional cushioning properties, it is often used as a packaging material and filler [21,22]. Research has focused mainly on cushioning applications, such as helmets designed with fractal structures, cushioning trays [23], cushion packaging made from thin shells of palm fiber [24], loofah sponge kitchenware, mattresses, and shoe soles. However, there are few applications in the area of mechanical structure. In particular, there is no related research on low-frequency mechanical vibration isolation structures. Therefore, this study investigated the biological characteristics of the loofah sponge through measurements and observations of its macroscopic dimensions and explored its mechanical properties and effects through experiments and simulations to examine the vibration isolation mechanism of the loofah sponge.

## 2. Materials and Methods

**Figure 1:**
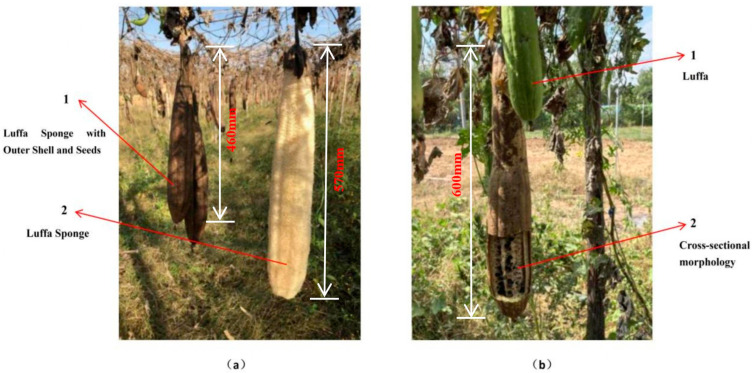
Loofah sponge. (**a**) Luffa Sponge with Outer Shell and Seeds 1 and Loofah Sponge 2. (**b**) Luffa 1 and Cross-sectional Morphology of Loofah 2.

The loofah sponge used in this experiment was purchased from a loofah sponge planting base in Nanyang, Henan Province. To ensure experimental consistency, all loofah sponges were of the same variety, grown in the same planting area and in the same planting year. The variety used is the locally cultivated three-hole soft loofah sponge as shown in Figure 1. Figure 1(a1) shows the loofah sponge after natural sun drying, with the hard outer shell, seeds, and film still intact, while Figure 1(a2) displays the loofah sponge after the removal of the hard outer shell and seeds. Figure 1(b1) shows the appearance of the loofah sponge’s cross-sectional cut.

### 2.1. Loofah Sponge Structure

**Figure 2:**
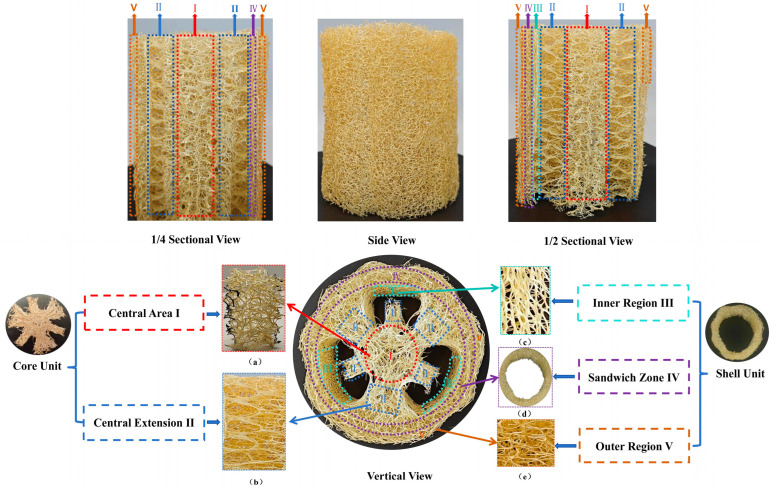
Macroscopic structure diagram of loofah sponge. Central Area I: (**a**) Central Structural Portion of Loofah Sponge in the Red-Highlighted Region. Central Extension II: (**b**) Hexagonal Porous Structure Connecting Central Area I and Inner Region III. Inner Region III: (**c**) Composed of Axially Aligned, Continuous Fiber Structure. Sandwich Zone IV: (**d**) Dense Fiber Structure Sandwiched Between Inner Region III and Outer Region V. Outer Region V: (**e**) The outermost layer characterized by lateral fiber structure.

Figure 2 illustrates the macroscopic structural diagram of the loofah sponge, which exhibits a complex, three-dimensional geometric arrangement with a regular distribution of multidirectional, porous shapes. The macroscopic structure is divided into five regional structures from the center to the outermost part: the central region I, the central extended region II, the inner region III, the interlayer region IV, and the outer region V. The central region I and the central extended region II together form the core unit, while the inner region III, the interlayer region IV, and the outer region V form the shell unit. The loofah sponge fibers in each corresponding region are labeled as fibers I, fibers II, fibers III, fibers IV, and fibers V. The central region I features a multilayered structure designed for seed storage, as shown in Figure 2a. This region is characterized by good resilience, a soft texture, and low axial rigidity. The central extended region II, as shown in Figure 2b, consists of interlaced layers along the axial direction and pores with a hexagonal shape. This larger pore is where the loofah sponge stores its seeds. The inner region III, as shown in Figure 2c, is composed of thick fibers arranged vertically and densely, resulting in a hard texture and high axial rigidity. The interlayer region IV (Figure 2d) connects the inner region III and the outer region V (Figure 2e) with more transverse fibers. The fiber hardness and diameter lie between those of the fibers in the outer region and the fibers in the round hole region. Finally, the outer region V is the outermost layer, consisting of the softest fibers arranged densely in a circumferential direction. In addition, three elliptical cavities are located between the core and shell units of the loofah sponge.

### 2.2. Specimen of Loofah Sponge

**Figure 3:**
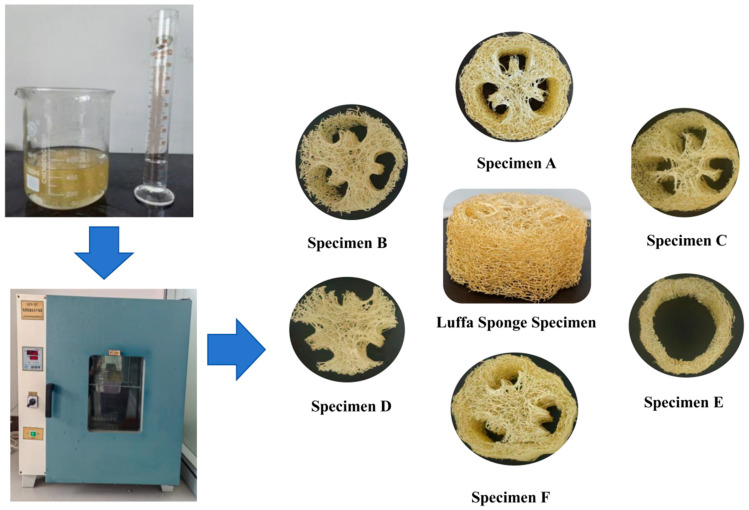
Specimen of loofah sponge.

The loofah sponge specimen was cleaned to remove seeds and any residual film from the center. A 40-mm high cylinder was then cut from the loofah sponge along the axial direction using a cutter. The sponge was immersed in distilled water to dissolve the film and wash away dust and other debris. Following this, the loofah sponge was placed in an oven set to 50 °C for 8 h to dry, producing the loofah sponge specimen, as shown in Figure 3. Specimen A was a complete loofah sponge specimen. Specimen B was a loofah sponge specimen with the outermost fibers removed (equivalent to the complete loofah sponge with area V removed). Specimen C was a loofah sponge specimen with the vertical fibers in the inner area removed (equivalent to the complete loofah sponge with area III removed). Specimen D was a core unit specimen (including the central region I and the central extended region II). Specimen E was a shell unit specimen (including the inner region III, the interlayer region IV, and the outer region V). Finally, specimen F was a specimen in which the core unit and shell unit were separated and then recombined.

### 2.3. Static Compression Test of Loofah Sponge

**Figure 4:**
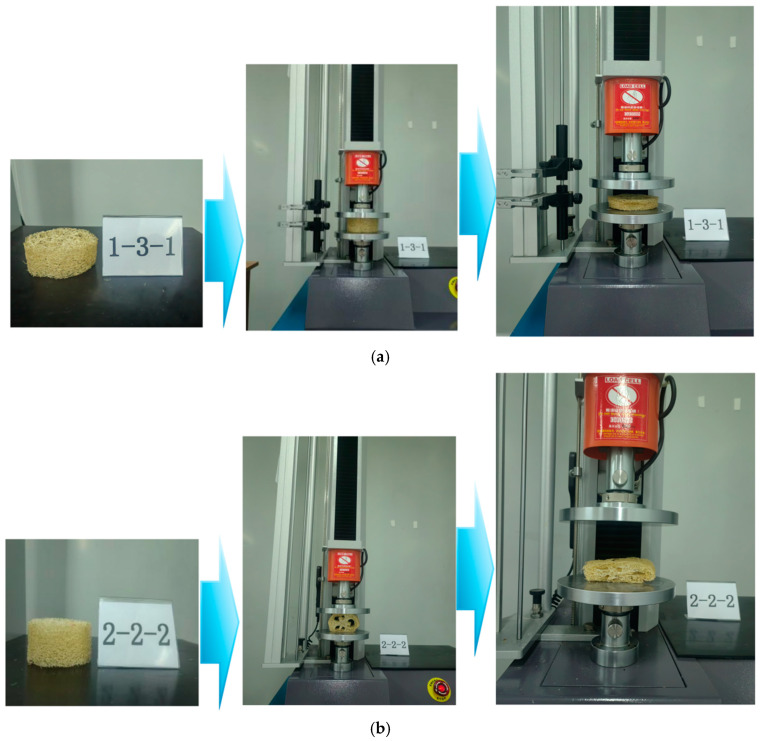
Schematic diagram of the loofah sponge compression test. (**a**) Schematic diagram of axial compression of loofah sponge. (**b**) Schematic diagram of radial compression of loofah sponge.

Density is a fundamental property of materials and significantly influences their structural performance. The fiber density of the different regions of a loofah sponge varies. Therefore, the average fiber density (ρ_s_) was calculated by dividing the mass of the loofah sponge specimen by its volume. The structural density (ρ_z_) was determined by dividing the cross-sectional area, which was calculated using the mass of the specimen and its maximum diameter. A high-strength steel testing instrument (Dongguan, Guangdong Co., Ltd. AL-3000 single-column computer system tensile testing machine) was used to perform a static compression test on loofah sponge specimens with varying densities. The specimen characteristics are summarized in Table 1. (Note: The actual density refers to the true density of the loofah sponge after all pores have been removed using the water drainage method; the structural density refers to the calculated density, which includes the pores; and the cross-sectional area refers to the actual cross-sectional area after the pores have been removed). The test was conducted at a speed of 0.1 mm/s, as shown in Figure 4.

To investigate the effect of the structure of different parts on the energy absorption and load-bearing capacity of the loofah sponge, we conducted three comparative tests on loofah sponge specimens using the controlled variables method. The specimens tested included the intact part, the part with region V removed, the part with region I removed, the part with region III removed, and the part with the core unit removed. A radial compression test was performed on the loofah sponge specimens (the test diagram is shown in Figure 4b) to investigate the effect of the fiber structure of the different regions on the radial mechanical properties of the loofah sponge.

### 2.4. Loofah Sponge Vibration Isolation Test

**Figure 5:**
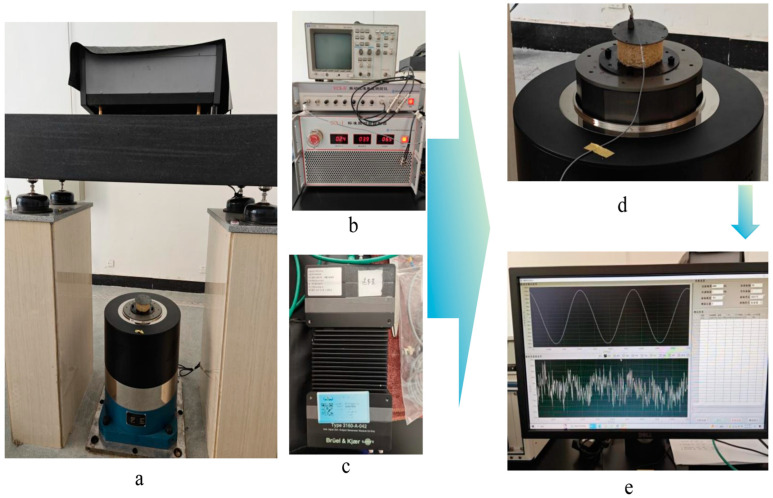
Vibration test platform. (**a**) Standard vibration testing platform. (**b**) Standard vibration controller. (**c**) Acoustic analyzer. (**d**) Accelerometer (**e**) Frequency response test software.

To explore the isolation vibration intensity characteristics of the loofah sponge, we constructed a vibration test platform, as shown in Figure 5. The platform consisted of a mechanical actuator that generated vibration excitation, a power control unit, an acoustic analyzer, an acceleration sensor, and frequency response test software. The mechanical actuator utilized a standard vibration test bench (operating frequency range of 0.005 Hz–1000 Hz, maximum displacement of 100 mm) to provide the vibration source. It also recorded the acceleration input to the test bench via a built-in acceleration sensor. The power control unit employed a standard vibration controller to regulate the magnitude of the exciting vibration. The acoustic analyzer detected noise interference during the test, while the acceleration sensor collected the acceleration output after isolation by the silk gauze. The frequency response test software processed and analyzed both the input and output accelerations. During the test, periodic vibration excitation was applied to the bottom of the prototype, and specific frequency points between 1 and 1000 Hz were selected for targeted testing.

**Figure 6:**
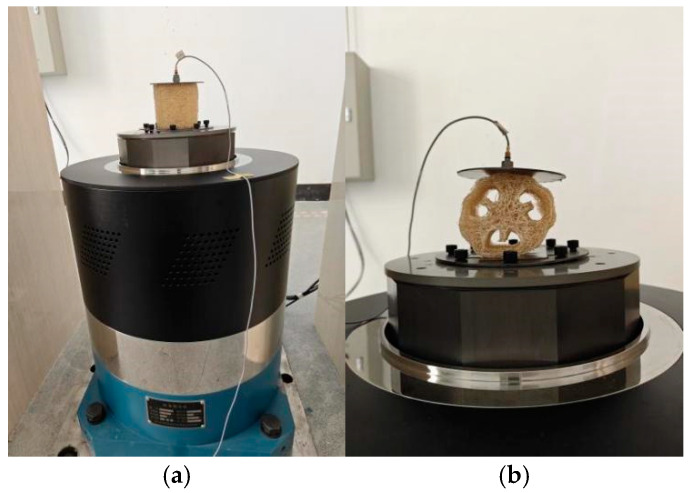
Schematic of the loofah sponge vibration isolation test. (**a**) Loofah sponge axial vibration isolation test. (**b**) Loofah sponge radial vibration isolation test.

To securely attach the loofah sponge specimen to the standard vibration test bench and measure the output acceleration on the upper surface, we designed and fabricated a 0.5 mm thick fixture with a diameter of 110 mm, made of Q235. The fixture was fastened to the standard vibration test bench using an Allen screw, and an acceleration sensor was placed on the outer surface of the upper plate to measure the output acceleration after vibration isolation by the loofah sponge network. As shown in Figure 6, vibration isolation tests were conducted on the complete loofah sponge network specimen in both the axial and radial directions, respectively.

### 2.5. Loofah Sponge Model Construction and Simulation

**Figure 7:**
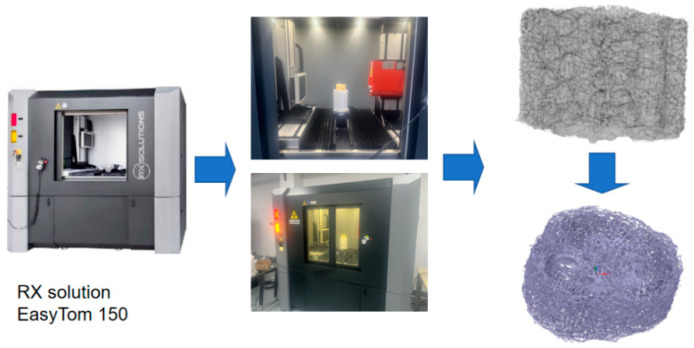
Industrial CT scan modelling.

Since the loofah sponge is a multipart structure, a French RX Solutions nano-CT system (with a resolution of 400 nm) was employed to further explore the coupling effect and mechanism of its main structure. The system utilized 3D X-ray computed tomography technology to scan the loofah sponge, as shown in Figure 7. The device’s accompanying software was then used to reconstruct a 3D model of the scanned loofah sponge, generating an STL format file.

**Figure 8:**
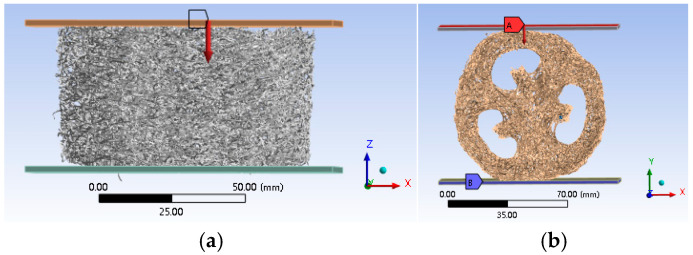
ANSYS finite element static compression diagram. (**a**) Axial compression along the direction of the arrow. (**b**) Radial compression along the direction of the arrow.

We imported the STL file into the finite element software ANSYS Workbench 2023 R1 to create a 3D model of the various components of the bionic model. The model was meshed using HyperMesh 2022 software, and the meshed model was subsequently imported into ANSYS Workbench. The meshed model was imported via the External Model module and analyzed statically using the Static Structural module to explore the stress and compression of each part of the complete loofah sponge specimen under a pressure of 130 N in the axial and radial directions, as shown in Figure 8.

**Figure 9:**
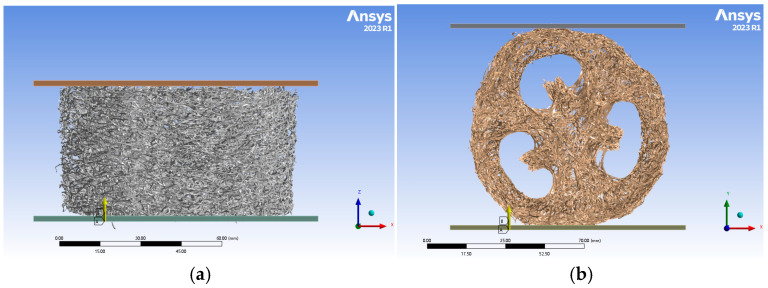
Schematic diagram of ANSYS finite element vibration analysis. (**a**) Axial vibration isolation test. (**b**) Radial vibration isolation test.

During the vibration test, high-frequency excitation of the equipment generated noise at high frequencies. The loofah sponge specimen was small, and when the noise level is high, it can significantly interfere with the test, thereby affecting the accuracy of the data. To ensure the reliability of the test results, we used a c-sound analyzer to detect noise. The range of the testing equipment, the measurable frequency range of the complete loofah sponge, was restricted to 1–750 Hz. To explore the vibration isolation behavior of the complete loofah sponge over a wider frequency range, we conducted a simulation analysis for both the axial and radial directions, with vibration excitation applied along the direction of the arrow, as shown in Figure 9. This simulation allowed for the direct input of acceleration at the lower plate without generating noise, enabling the study of the loofah sponge specimen across a broader frequency range, unaffected by equipment limitations. In the simulation model, the loofah sponge was fixed between two metal plates, and connected in a bound manner. Acceleration was applied at the lower plate, and the acceleration at the upper plate was then measured. The frequency range was 1 Hz–2000 Hz.

## 3. Results and Discussion

The vibration isolation performance of the loofah sponge results from a combination of energy absorption and vibration isolation. Therefore, the tests and simulations focused on the structural and fiber characteristics of each region of the loofah sponge (Figure 2) to analyze their contributions to energy absorption and vibration isolation and to examine their impact on vibration isolation performance.

**Figure 10:**
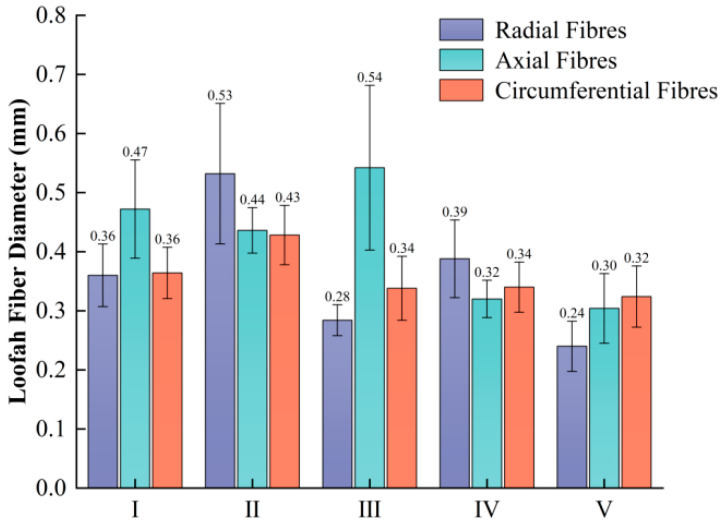
Measurement of the diameter of the loofah sponge fibers in different areas.

The central area (area I) and the connecting area (area II) primarily serve to store seeds. Their structure consists of numerous polygonal cavities with large pores arranged in layers. Moving from the center to the periphery, the average pore size gradually decreases (Figure 2), while the fiber diameter exhibits a characteristic alternating pattern. Specifically, the average diameter of the axial fibers is the largest in region III, while the average diameter of the radial and circumferential fibers is largest in region II (Figure 10). This fibrous structure, with different characteristics, coupled with multidirectional rigidity and flexibility, allows the sponge to exhibit excellent and stable overall mechanical properties.

### 3.1. Absorbent Energy Properties of Loofah Sponge

**Figure 11:**
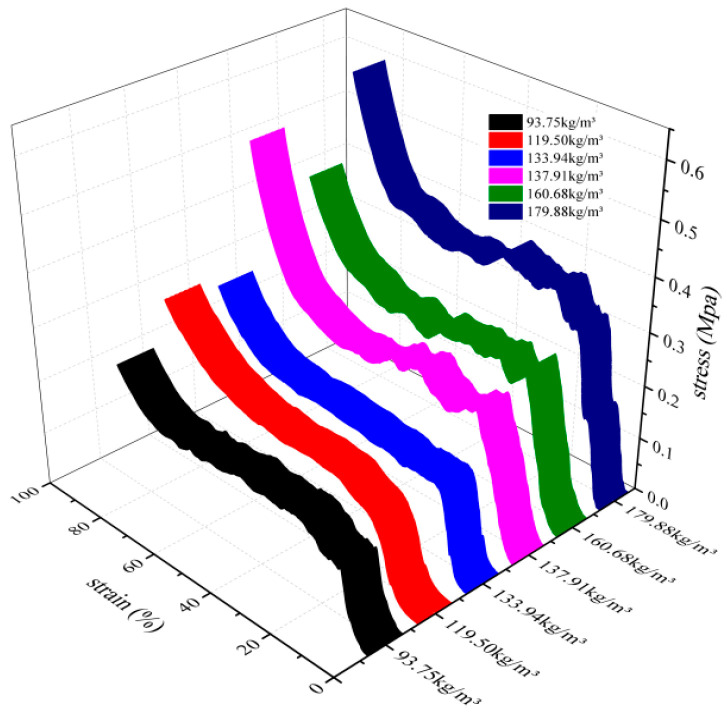
Stress–strain curves for axial compression of loofah sponge specimens with different densities.

As shown in the three-dimensional stress-strain curve in Figure 11, the sponge with varying densities undergoes three stages during static compression: the elastic stage, the yielding stage, and the densification stage [25]. As the density of the sponge increases, the stress–strain curve also increases. According to Equation (1), the energy absorbed per unit volume of the sponge structure, denoted as e, can be calculated. The value of e is equivalent to the area enclosed by the stress–strain curve, the abscissa, and the ordinate axes. This study focused on the energy absorption characteristics of the loofah sponge structure and, therefore, excluded the densification stage that occurs after the compression of the loofah sponge fibers. Instead, a 40 mm high loofah sponge specimen was compressed by 60% under a static load (within the yielding stage range), as shown in Table 2. The energy absorbed per unit volume when a 40 mm-high loofah sponge specimen is compressed 60% of its height. The mass of loofah sponge is smaller than that of Coscinoderma sp., and its energy absorption per unit volume is more than three times that of Coscinoderma sp., demonstrating excellent energy absorption performance [26,27]. The six groups of specimens demonstrate that as the density of the loofah sponge increases, the energy absorbed per unit volume also increases.

(1) $$e=\int_{0}^{\varepsilon }\sigma d\varepsilon$$

where e is the energy absorbed per unit volume during compression of the specimen; σ is the stress; and ε is the strain.

As shown in Figure 11, the stress exhibits multiple peaks and troughs beyond the upper yield point. This behavior results from the combined effects of the multilayer structure in the central region and the axial pores present in each section. Based on the curve data and the testing process, the distance between the peaks after the upper yield point closely corresponds to the layer spacing in the central area. As the height of the compression axial dimension decreases and the overall density of the structure increases, the energy absorbed per unit of compression stroke also increases. The energy absorbed during the compression of the specimen E Equation (2) is equivalent to the work done by the compression load, as follows:

(2) $$E=\int_{0}^{x}Fdx=\int_{0}^{\varepsilon }\mathrm{SH}\sigma d\varepsilon =\mathrm{SH}\int_{0}^{\varepsilon }\sigma d\varepsilon$$

where *E* is the energy absorbed during compression of the specimen; *F* is the positive pressure; *x* is the displacement of downward compression; S is the cross-sectional area of the specimen subjected to radial compression; and H is the axial height of the specimen.

**Figure 12:**
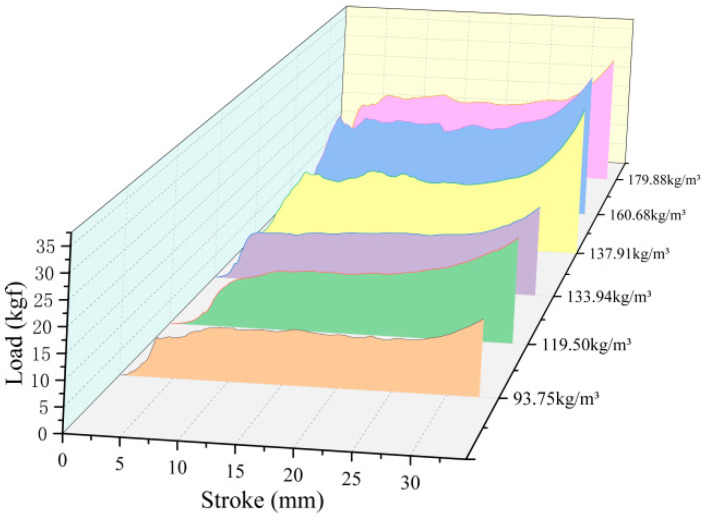
Graph depicting the axial load and deformation of loofah sponge. (The different colors in the figure represent the force and displacement curves at different densities.).

The load-deformation curve of the loofah sponge is shown in Figure 12. The energy absorption of the loofah sponge specimen was calculated using the integral function of the mathematical module in Origin 2021 software, with the compression stroke set to 24 mm (i.e., 60% of the height), as shown in Table 3. The results show that the specific energy absorption of loofah sponge is comparable to that of aluminum foam and most polymer foams, demonstrating excellent energy absorption performance, and the energy absorption of the loofah sponge’s overall structure tends to decrease as the density decreases; however, it is not solely determined by the density [28,29]. Other factors also influence energy absorption, which may be attributed to variations in the structural sizes of the fibers across different regions of the sponge. Therefore, a comparative analysis of the structure in each region is necessary.

**Figure 13:**
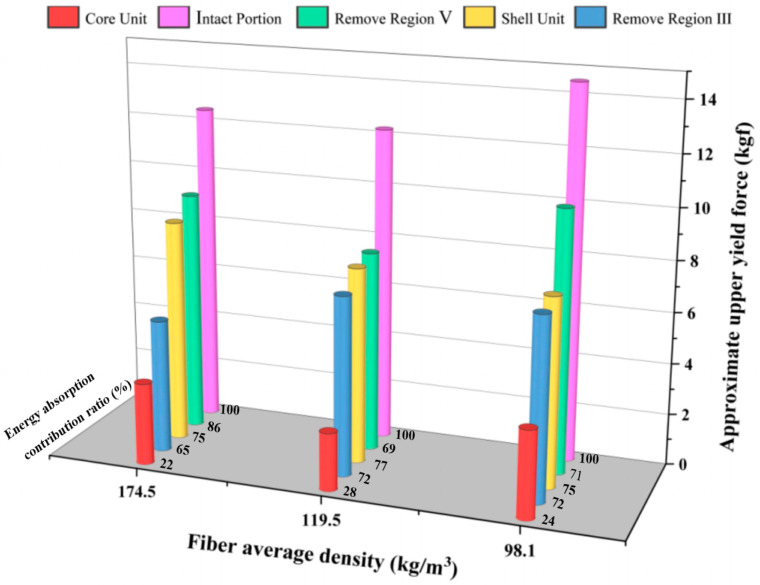
Comparative test of axial compression of different regions of loofah sponge.

Figure 13 shows the results of a comparative test on the axial compression of different loofah sponge structures. The energy absorption contribution rate is obtained by dividing the energy absorbed in each region by the total energy absorbed during compression of the entire loofah sponge. The results show that during axial compression, the bearing capacity of the loofah sponge in the elastic range follows the order: intact portion > remove region V > shell unit > remove region III > core unit. This indicates that the vertical fibers in part III play a significant role in supporting the structure, while the fibers in the outermost region V offer the least resistance to axial force. The intact loofah sponge exhibits obvious force yielding, including both upper and lower yielding forces. However, once the fibers in region III are removed, the entire loofah sponge no longer shows obvious yielding, and its axial stiffness is significantly reduced. In particular, when region I is compressed independently, the stress–strain curve shows a clear continuous hardening, further reflecting the critical role of the vertical fibers in region III in improving the overall stiffness. The structure of the central region I exhibits minimal change within a certain range in the slope of the force–displacement curve during compression under external forces, which resembles the behavior of a spring under external force. The energy absorption of the loofah sponge with a density of 119.5 kg/m^3^ in five different structures was calculated using the integral function of the Origin 2021 software’s mathematical module, under axial compression to 60% of its height (i.e., 24 mm). As shown in Table 4 and Figure 13, the results indicate that the shell unit structure is the primary contributor to the axial energy absorption, while the core unit structure exhibits the lowest energy absorption.

**Figure 14:**
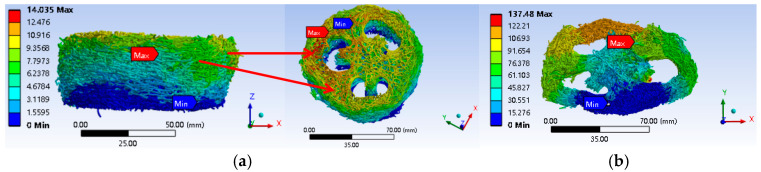
Compression deformation cloud map of loofah sponge. (**a**) Axial compression deformation cloud map. (**b**) Radial compression deformation cloud map.

**Figure 15:**
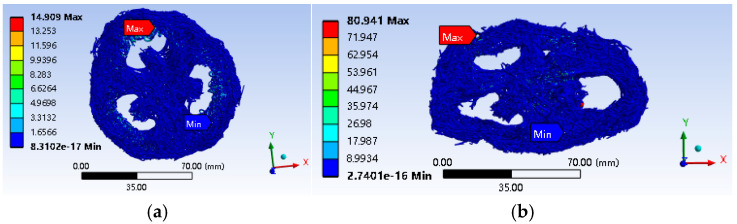
Compressive stress cloud diagram of loofah sponge. (**a**) Axial compression stress cloud map. (**b**) Radial compression stress cloud map.

Figure 14 and Figure 15 present the deformation and stress cloud diagrams of a loofah sponge subjected to a static load of 130 N. From these figures, it is evident that after compression, the deformation in the upper layer of most areas along the direction of the applied force is significantly greater than that in the lower layer, with a maximum deformation of 14.035 mm. The deformation progressively decreases from top to bottom along the force direction. This phenomenon can be attributed to the interaction between the layers in the sponge’s layered structure, which causes the stress to decrease gradually as it transfers between layers. Figure 15a shows that the shell unit experiences the highest average stress, with a peak value of 12.291 MPa. This reflects the relatively continuous structure and high stiffness of the shell unit along the axial direction. Figure 14b shows the deformation cloud diagram during radial compression. Under radial compression, the shell unit at the top of the specimen is the first to collapse, while the core unit exhibits the smallest deformation at the same height level. The average stress in this region is also the highest, as shown in Figure 15b. The maximum stress value is 80.941 MPa, indicating that the core has greater radial stiffness and plays a more significant role in supporting the structure radially. This indicates that the core unit contributes more to the stability of the radial structure and less to the axial stability.

As shown in Figure 11, the energy absorption per unit volume increases with the density of the loofah sponge specimens across the six test groups. During the force yield stage, the loofah sponges can absorb between 90.3 and 188.4 kJ of energy when compressed to a height of 24 mm (Table 2). Additionally, a single loofah sponge can absorb 2.13 to 4.06 J of energy (Table 3), demonstrating that a loofah sponge is not only an excellent energy-absorbing material [30,31], but also has an effective energy-absorbing structure. The shell unit structure plays a major role in axial energy absorption (Table 4). The fibers in shell unit part III extend throughout the entire length of the melon and provide the greatest support (Figure 13). These fibers bear the most stress in the axial direction (Figure 15b). The core unit limits the deformation of the shell unit and serves as a stabilizing structure (Figure 14).

### 3.2. Vibration Isolation Properties of Loofah Sponge

*Luffa cylindrica*, which ripens over a period of seven months from September to March, is often subjected to horizontal radial wind and vertical axial rain in its growing environment. Wind-induced vibrations of the loofah sponge primarily include structural vibrations, vortex-induced vibrations, and high-frequency forced vibrations generated by the rigid outer skin. According to Strouhal’s formula (3), the frequencies generated by the loofah sponge in the typical wind speed range (0–30 m/s) are approximately 0–200 Hz. Due to the relatively light, thin, and elastic nature of the creases and damaged areas of the loofah sponge’s hard rind, they are affected by local aerodynamic forces, which in turn generate fine high-frequency vibrations. These manifest as localized high-frequency tremors, especially when the wind speed approaches 30 m/s. At this point, the loofah sponge exhibits local high-frequency vibrations of lower amplitude, with high-frequency vibration waves transmitted from the hard outer skin to the interior of the loofah sponge. In addition, when raindrops strike the hard skin of the loofah sponge, they generate impact vibrations, resulting in high-frequency vibrations with a frequency range of several hundred to several thousand hertz.

(3) $$f=\frac{S\cdot V}{L}$$

In Equation (3), *f* is the vortex shedding frequency; *S* is the Strouhal number; *V* is the wind speed, and *L* is the diameter of the loofah sponge.

As shown in half-section B of the mature loofah sponge cross-section in Figure 2, the seeds stored at the center of the loofah sponge have not fallen out despite prolonged exposure to radial wind and axial raindrops. This suggests that, from a biological perspective, the loofah sponge likely possesses excellent vibration isolation properties.

**Figure 16:**
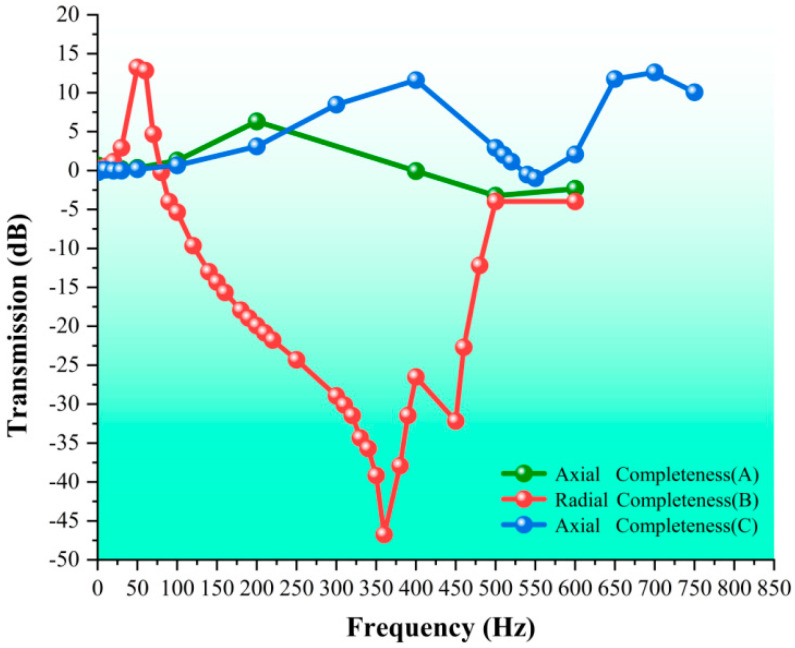
Frequency response curve of loofah sponge under sinusoidal vibration excitation.

**Figure 17:**
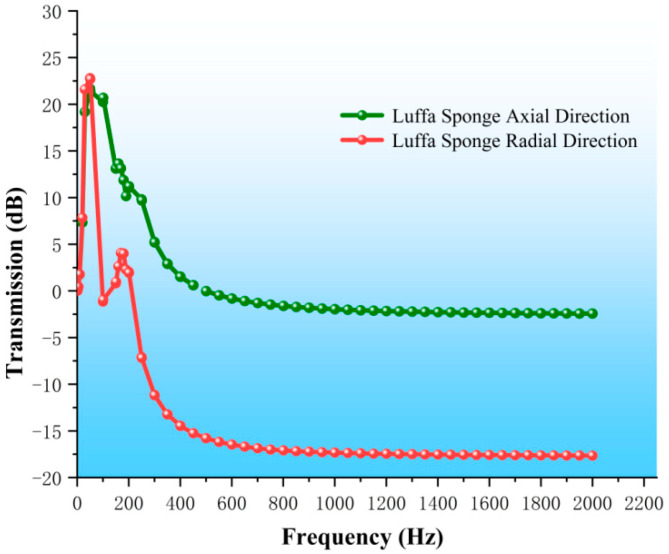
Simulated frequency response curve of loofah sponge.

Vibration isolation tests were conducted on a complete loofah sponge specimen (i.e., specimen A) in both the axial and radial directions. The frequency response curves under sinusoidal vibration excitation are shown in Figure 16 and Figure 17. Axial integrity (A) refers to the vibration isolation test performed on a complete loofah sponge specimen in the axial direction. A resonance point was observed at a frequency of approximately 200 Hz, with the vibration isolation effect beginning at 400 Hz. Radial integrity (B) corresponds to the vibration isolation test of a complete loofah sponge specimen in the radial direction. The radial vibration isolation test was conducted on the intact (B) loofah sponge specimen. The resonance frequency was approximately 30 Hz, and the vibration isolation effect began at 80 Hz. The vibration isolation effect was best at 360 Hz, with a vibration transmission of −46.74 dB, which can isolate 99.54% of the vibration effect. Finally, the axial intact (C) specimen refers to a high-density coarse-fiber loofah sponge. The vibration isolation test was conducted in the axial direction. We observed a vibration isolation effect only at approximately 540 Hz, which was not good.

The test results indicate the following: (1) the complete loofah sponge exhibits a markedly superior radial vibration isolation effect compared to axial vibration isolation within the frequency range of 1–750 Hz, as measured in this study; (2) the loofah sponge demonstrates effective low-frequency and medium-frequency vibration isolation effects in the radial direction, along with a broad frequency range of vibration isolation; (3) although high-density loofah sponge absorbs more energy per unit volume, its vibration isolation performance is inferior to that of low-density loofah sponge. This suggests that the primary vibration isolation effect of the loofah sponge specimen is attributed to its structure rather than the material.

The simulated frequency response curve of the loofah sponge is shown in Figure 17. The frequency range of the loofah sponge model with axial vibration isolation is from 500 Hz to 2000 Hz, with a maximum vibration transmission of −2.43 dB. The vibration isolation effect is not ideal. In contrast, the frequency range of the loofah sponge model with radial vibration isolation extends from 249 Hz to 2000 Hz, with a maximum vibration transmission of −17.64 dB. This effectively isolates 86% of the vibration, demonstrating an excellent vibration isolation effect.

The results of the simulation analysis are consistent with the experimental results in terms of trends, although the vibration isolation effect is not as pronounced as in the experimental data. This suggests that the finite element analysis is effective and accurately reflects the general trend. However, it cannot fully account for the self-contact and frictional interactions between the loofah sponge fibers during testing. As a result, the simulation data for the vibration isolation test are lower than the experimental data, and unlike in the physical test, no multiple resonance points occur due to these interactions. The deviation remains within a predictable range.

The simulation results show that: (1) in the frequency range of 1–2000 Hz, the loofah sponge exhibits excellent vibration isolation effect in the radial direction across both low and high frequencies; (2) in the axial direction, the loofah sponge demonstrates vibration isolation only in the medium and high-frequency ranges, and the effect is less pronounced.

**Figure 18:**
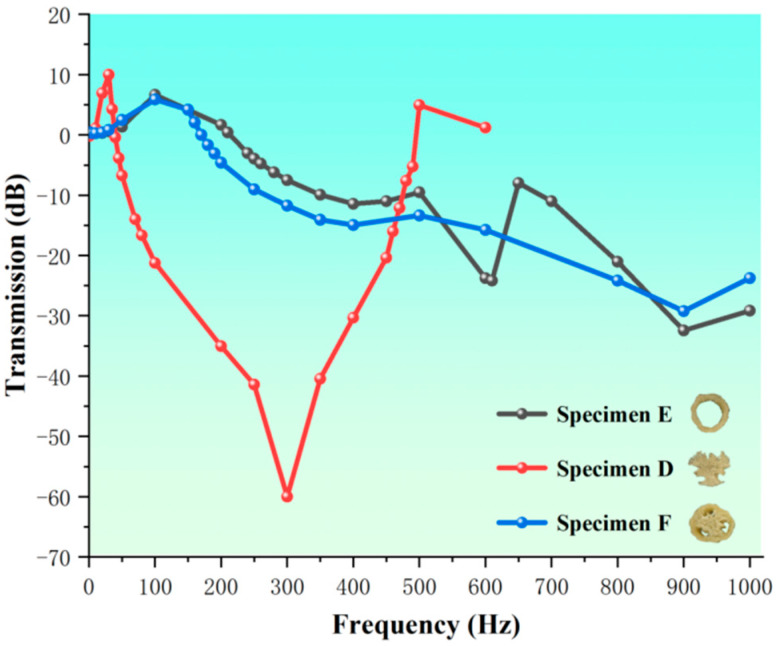
Vibration transmission diagram of a loofah sponge specimen excited by sinusoidal vibration.

To further explore the mechanism of vibration isolation of loofah sponges, we examined three types of specimens: the core unit (specimen D), the shell unit (specimen E), and a combination of the core and shell units that were not connected (specimen F). The results of the axial vibration isolation test are shown in Figure 18. The results indicate that the core unit structure (specimen D) provides the best vibration intensity isolation effect. This specimen exhibits a vibration isolation effect starting at 40 Hz, with a vibration transmission of −60 dB at 300 Hz, isolating 99.9% of the vibration intensity. The effectiveness of vibration isolation is ranked from highest to lowest as follows: specimen D > specimen F > specimen E. Specimen D consists of a central region I with low axial stiffness, which provides a good elastic effect, and an outer extended region II that improves axial support. This combination of a soft interior and a rigid exterior greatly improves vibration isolation performance. Specimen E, a complete loofah sponge specimen with the core unit removed, shows vibration isolation starting at 240 Hz. At a frequency of 900 Hz, the vibration transmission is −32.4 dB, isolating 97.63% of the vibration intensity. Without the strong radial support provided by the core unit, specimen E is more prone to radial deformation, which can dissipate vibration energy and isolate vibration intensity. Specimen F shows vibration isolation starting at 170 Hz, with a vibration transmission of −29.21 dB at 900 Hz, isolating 96.53% of the vibration intensity. Since fibers III extend axially through the core and the structural stiffness of the shell unit is much greater than that of the core, the vibration wave primarily travels along the shell unit, including the inner layer area III in the axial direction. The connection between the core and shell units improves the stability of the overall structure but also limits the deformation of the shell unit. As a result, while the unit volume becomes better able to absorb energy, its ability to isolate vibration intensity is reduced. Compared to the complete loofah sponge specimen, the vibration isolation capabilities of specimens E and F are greatly enhanced due to the absence of radial constraints. Specimen D undergoes greater deformation when subjected to external vibration, facilitating the absorption and reduction of vibration energy. The large pores within the structure also provide a good capacity for stress distribution, which can more effectively reduce local stress concentrations caused by vibration and thereby enhance the overall vibration isolation performance under axial vibration.

The loofah sponge is composed of two parts: the core unit structure and the shell unit structure. The core unit structure consists of the central area I and the connection area II, while the shell unit structure is made up of the inner area III, the interlayer area IV, and the outer area V. The structures serve distinct functional purposes in different directions. In the axial direction, the stiffness of the core unit structure is low, primarily serving to isolate vibration, while the stiffness of the outer unit structure is high, primarily serving to absorb energy and provide support. In the radial direction, the roles are reversed: the core unit, with its high stiffness, stabilizes the structure and limits deformation, while the outer unit structure, with its lower stiffness, absorbs less energy and primarily serves to isolate vibrations.

Overall, the structure of the loofah sponge exhibits excellent low-frequency vibration isolation across multiple parts. The isolated core unit structure provides the best axial vibration isolation. This is due to the central area I, which has functional characteristics similar to a spring and has low stiffness, while the connecting area II, resembling a multilayer hexagonal structure, acts as a nonlinear support with high stiffness. The combination of the soft and hard materials causes the central area I to be more prone to substantial deformation under axial force or vibration. This, in turn, provides a supporting force from the stiff connecting area II. The shell unit structure in the radial direction delivers the best vibration isolation effect. The entire shell unit takes the form of a thin cylinder, with the stiffness of the fibers gradually decreasing from the inner to the outside, exhibiting a certain degree of negative Poisson’s ratio, which enhances its vibration isolation properties. As shown in Figure 18, the integration of the core unit and shell unit structures provides both axial and radial vibration isolation, offering valuable insights for the design of multidirectional low-frequency vibration isolation devices.

## 4. Conclusions

This study systematically verified the superiority of the loofah sponge structure in terms of vibration isolation performance through both experimental and simulation analysis, with a focus on its remarkable energy absorption capacity and effective isolation effect on vibration intensity. Additionally, the vibration isolation mechanism of the multiple structural units with rigid–flexible coupling within the loofah sponge structure was thoroughly examined, providing theoretical insights into its unique vibration isolation characteristics. Based on the characteristics of the loofah sponge structure, the loofah sponge is divided into two components: the core unit and the shell unit. The structure is further divided into five regional layers, from the interior to the exterior. Measurement and analysis of the fiber size revealed that the size and structure of the loofah sponge fibers exhibit the regular characteristics of rigid–flexible coupling. In static compression tests of six complete loofah sponge specimens, the loofah sponge was found to absorb between 90.3 and 188.4 kJ of energy per unit volume when compressed by 24 mm during the force-yielding stage. A single loofah sponge absorbs between 2.13 and 4.06 J of energy, demonstrating good energy absorption characteristics. Regarding energy absorption, the shell unit structure plays a major role in the axial direction. The fibers in the shell unit extend along the entire length of the sponge, making it the most supportive. The core unit limits deformation in the shell unit structure and plays a role in stabilizing the structure. The structure of the various parts of the loofah sponge exhibits excellent vibration isolation effect: the loofah sponge structure begins to show radial vibration isolation at 80 Hz, with peak vibration isolation effect at 360 Hz, achieving a vibration transmission reduction of −46.74 dB. The core unit exhibits the best axial vibration isolation effect, beginning at 40 Hz, with a vibration transmission of −60 dB at 300 Hz. The shell unit structure demonstrates axial vibration isolation beginning at 240 Hz and demonstrates an axial vibration isolation effect. The vibration transmission is −32.4 dB at a frequency of 900 Hz. The combination of the core unit and the shell unit structure achieves an axial vibration isolation effect starting at 170 Hz, with a vibration transmission of −29.21 dB at 900 Hz. This study revealed the vibration isolation mechanism of the loofah sponge structure, providing theoretical support for its application in vibration isolation design. Furthermore, our findings provide new insights for the biomimetic design of small-size, low-frequency vibration isolation devices based on the silk-melon structure, expanding design methodologies and the potential applications in the field of low-frequency vibration isolation.

## Figures and Tables

**Table 1:** Characteristic parameters of the test specimen.

Actual Density ρs (kg/m^3^)	Structural Density ρz (kg/m^3^)	Height H (mm)	Diameter of the Dross-Section D (mm)	Cross-Sectional Area S (cm^2^)	Type of Specimen	Compression Direction
93.75	13.22	40	73.00	5.90	Specimen A	Axial
119.50	12.45	40	89.10	9.72	Specimen A	Axial
133.94	12.08	40	92.04	6.00	Specimen A	Axial
137.91	14.71	40	85.50	6.13	Specimen A	Axial
160.68	17.26	40	93.50	7.38	Specimen A	Axial
179.88	21.12	40	73.64	4.32	Specimen A	Axial
174.48	15.39	40	89.70	5.58	Specimen A	Axial
98.13	12.02	40	77.32	5.75	Specimen A	Axial
161.91	14.73	40	97.20	9.72	Specimen A	Radial
89.76	10.79	40	74.56	5.25	Specimen B	Axial
146.22	12.70	40	81.90	8.19	Specimen B	Radial
277.21	16.95	40	69.20	2.30	Specimen B	Axial
91.10	9.86	40	82.24	5.75	Specimen B	Axial
136.65	10.65	40	93.72	9.38	Specimen C	Radial
94.74	9.86	40	80.16	5.25	Specimen C	Axial
106.26	10.55	40	89.52	6.25	Specimen C	Axial
191.65	13.35	40	88.90	4.32	Specimen C	Axial
55.50	7.32	40	65.00	4.38	Specimen D	Axial
132.42	14.06	40	53.36	2.38	Specimen D	Axial
52.90	7.19	40	63.08	4.25	Specimen D	Axial
82.30	8.06	40	65.00	6.50	Specimen D	Radial
167.56	9.26	40	97.50	9.75	Specimen E	Radial
219.15	11.59	40	88.80	3.28	Specimen E	Axial
111.46	8.49	40	92.54	5.13	Specimen E	Axial
86.80	8.41	40	85.00	5.50	Specimen E	Axial

**Table 2:** Energy absorption per unit volume of loofah sponge with different densities.

Actual Density ρs (kg/m^3^)	179.88	160.68	137.91	133.94	119.50	93.75
Energy absorption per unit volume(kJ/m^3^)	188.40	137.5	127.70	99.40	91.30	90.30

**Table 3:** Energy absorption of different density loofah sponge specimens and specific energy absorption.

Actual Density ρs (kg/m^3^)	179.88	160.68	137.91	133.94	119.50	93.75
Energy absorption (J)	3.77	4.06	3.13	2.39	2.38	2.13
Specific Energy Absorption (MJ/kg)	1.048	0.857	0.926	0.744	0.766	0.963

**Table 4:** Energy absorption per unit volume of different regions of loofah sponge.

Loofah Sponge Specimen Type	Intact Portion	Remove Region V	Remove Region III	Shell Unit	Core Unit
Energy absorbed (kJ)	91.3	63.7	66.9	72.3	38.8

## Data Availability

The data that support the findings of this study are available upon reasonable request from the corresponding author. The data are not publicly available because of privacy or ethical restrictions.
